# Core Microbiota in Agricultural Soils and Their Potential Associations with Nutrient Cycling

**DOI:** 10.1128/mSystems.00313-18

**Published:** 2019-03-26

**Authors:** Shuo Jiao, Yiqin Xu, Jie Zhang, Xin Hao, Yahai Lu

**Affiliations:** a College of Urban and Environmental Sciences, Peking University, Beijing, People’s Republic of China; Michigan State University

**Keywords:** agricultural ecosystems, continental atlases, core microbiota, multinutrient cycling

## Abstract

Revealing the ecological roles of the core microbiota in community maintaining and soil nutrient cycling is crucial for understanding ecosystem function, yet there is a dearth of continental-scale studies on this fundamental topic in microbial ecology. Here, we collected 251 soil samples from adjacent pairs of maize and rice fields at a continental scale in eastern China. We revealed the major ecological roles of the core microbiota in maintaining complex connections between bacterial taxa and their associations with belowground multinutrient cycling. By identifying the habitat preferences of the core microbiota, we built a continental atlas for mapping the spatial distributions of bacteria in agro-soils, which helps forecast the responses of agricultural ecosystems to anthropogenic disturbance. The multinutrient cycling index for maize and rice soils was related to bacterial *α*-diversity and *β*-diversity, respectively. Rice soils exhibited higher bacterial diversity and closer bacterial cooccurrence relationships than maize soils. In contrast to the macro- or microecological latitudinal richness patterns in natural terrestrial ecosystems, the bacteria in maize soils showed higher richness at high latitudes; however, this trend was not observed in rice soils. This study provides a new perspective on the distinct bacterial biogeographic patterns to predict the ecological roles of the core microbiota in agro-soils and thus helps manage soil bacterial communities for better provisioning of key ecosystem services.

**IMPORTANCE** Disentangling the roles of the core microbiota in community maintaining and soil nutrient cycling is an important yet poorly understood topic in microbial ecology. This study presents an exploratory effort to gain predictive understanding of the spatial atlas and ecological roles of the core microbiota. A systematic, continental-scale survey was conducted using agro-soils in adjacent pairs of maize (dryland) and rice (wetland) fields across eastern China. The results indicate that the core microbiota play major ecological roles in maintaining complex connections between bacterial taxa and are associated with belowground multinutrient cycling. A continental atlas was built for mapping the bacterial spatial distributions in agro-soils through identifying their habitat preferences. This study represents a significant advance in forecasting the responses of agricultural ecosystems to anthropogenic disturbance and thus helps manage soil bacterial communities for better provisioning of key ecosystem services—the ultimate goal of microbial ecology.

## Abstract (precis)

Disentangling the roles of the core microbiota in community maintaining and soil nutrient cycling is an important yet poorly understood topic in microbial ecology. This study presents an exploratory effort to gain predictive understanding of the spatial atlas and ecological roles of the core microbiota. A systematic, continental-scale survey was conducted using agro-soils in adjacent pairs of maize (dryland) and rice (wetland) fields across eastern China. The results indicate that the core microbiota play major ecological roles in maintaining complex connections between bacterial taxa and are associated with belowground multinutrient cycling. A continental atlas was built for mapping the bacterial spatial distributions in agro-soils through identifying their habitat preferences. This study represents a significant advance in forecasting the responses of agricultural ecosystems to anthropogenic disturbance and thus helps manage soil bacterial communities for better provisioning of key ecosystem services—the ultimate goal of microbial ecology.

## INTRODUCTION

Soil microorganisms, representing one of the largest biodiversity reservoirs, participate in a variety of ecological processes in terrestrial ecosystems (1–3). For example, microorganisms undertake soil decomposition and mediate C, N, S, and P biogeochemical cycles (2, 3). In general, ecosystems perform multiple simultaneous functions and services (ecosystem multifunctionality), which depend on the ecological roles of the organisms living within a given area (4). Existing research on the links between biodiversity and ecosystem multifunctionality has mainly focused on plants over the past 2 decades (5–7). The diversity of soil organisms is known to promote ecosystem multifunctionality under controlled experimental conditions (3, 8), yet relationships can differ geographically due to spatial variation in abiotic factors (9). At large spatial scales, positive relationships are observed between microbial diversity and ecosystem multifunctionality in natural habitats (10), which may be primarily mediated by climate (9). Agricultural fields are typical human-managed terrestrial ecosystems essential for global food supply. Due to long-term agricultural land uses, soil physicochemical properties and ecosystem processes may vary compared with those in natural ecosystems, resulting in distinct microbial community assemblage patterns (11). Nutrient cycling is the most important agricultural ecosystem process for crop yields and supporting human welfare. Therefore, understanding the important factors involved in soil functionality linked to nutrient cycling is critical for managing human-dominated ecosystems. Currently, we still have limited knowledge on the relationships between microbial diversity and soil multiple-nutrient cycling in agricultural ecosystems, especially across different environments at continental scales.

In a particular type of habitat, a suite of members is broadly distributed among microbial communities at different locations, defined as the core microbiota (12, 13). Discovering such a core microbiota is critical to understand the assemblage and stability of microbial communities (12). Recently, a global atlas of the dominant soil bacteria in natural ecosystems was built, and a “most wanted” list was narrowed down to improve our knowledge of the spatial distributions of soil bacteria and their contributions to ecosystem functioning (14). However, we still lack a predictive understanding of the ecological attributes of individual bacterial taxa in agricultural ecosystems and their environmental preferences, traits, and metabolic capabilities. In addition, whether there exists a core microbiota of bacterial communities that are abundant and ubiquitous across agricultural soils from sites located far apart and, if so, whether this plays a major ecological role remain critical issues to resolve if we are to advance our understanding of soil bacterial community assemblage. Given that soil microorganisms rank among the most abundant and diverse groups of organisms on Earth, it is challenging to clarify their specific ecological attributes and contributions to ecosystem processes (14, 15). Moreover, agricultural ecosystems can be separated into wetland (e.g., paddy) and dryland (e.g., maize). Due to the differences in oxygenation, irrigation management, and agronomic practices, microbial diversity patterns are likely to be different in wetland and dryland soils. Presently, the fundamental processes underlying microbial biogeographic patterns remain poorly understood in these two distinct agricultural ecosystems, and this impedes our ability to predict agricultural ecosystem responses to current and future environmental changes.

Maize and rice are globally important crops, producing most of the world’s agricultural calories (16). Both of the crops are widely cultivated across China, making them suitable models for assessing the above-mentioned broad-scale questions. Here, we present a large-scale soil survey conducted across eastern China, including 126 maize and 125 rice fields under long-term cultivation. Most soil samples were collected from adjacent pairs of maize and rice fields at the same time to allow us to probe the influence of spatial scale and climatic factors on microbial diversity patterns. We analyzed bacterial diversity in soil samples by sequencing 16S rRNA genes and calculated a soil multinutrient cycling index based on measurements of edaphic variables associated with nutrient cycling. The aims of the present study were to (i) identify the core microbiota and construct a continental atlas for prediction of spatial distribution of soil bacteria in maize and rice fields and (ii) explore the ecological roles of the core microbiota in maintaining the connections between bacterial taxa and their potential associations with soil multinutrient cycling. Our findings provide insight into the spatial distributions and ecological roles of the core microbiota in agricultural ecosystems and thus help manage soil bacterial communities for better provisioning of key ecosystem services.

## RESULTS

### Bacterial diversity patterns in maize and rice soils.

Across all samples (see Fig. S1A in the supplemental material), we observed substantial differences in edaphic properties between maize and rice soils (see Fig. S2 in the supplemental material), although most soil samples were collected from adjacent pairs of fields. Cation exchange capacity (CEC), organic matter (OM), dissolved organic carbon (DOC), total nitrogen (TN), ammonium (NH_4_-N), microbial biomass carbon (MBC), microbial biomass nitrogen (MBN), total sulfur (TS), available sulfur (AS), and available iron (AFe) were significantly higher in rice fields. In contrast, nitrate (NO_3_-N), total phosphorus (TP), and available potassium (AK) were higher in maize soils.

10.1128/mSystems.00313-18.2 FIG S1 (A) Location of the 133 sampling sites of agricultural soils in eastern China, including 118 pairwise sites (both), eight maize single sites (maize), and seven rice single sites (rice). (B) Nonmetric multidimensional scaling ordination showing different bacterial β-diversity patterns of the agricultural soil samples. Download FIG S1, PDF file, 0.1 MB. Copyright © 2019 Jiao et al. 2019 Jiao et al. This content is distributed under the terms of the Creative Commons Attribution 4.0 International license.

10.1128/mSystems.00313-18.3 FIG S2 Variations in edaphic properties between maize and rice soils. Blue asterisks indicate the properties significantly higher in rice soils (*, *P* < 0.05; **, *P* < 0.01; ***, *P* < 0.001; Wilcoxon rank sum test); orange asterisks indicate the properties significantly higher in maize soils. DOC, dissolved organic carbon; OM, organic matter; MBC, microbial biomass carbon; TN, total nitrogen; AN, available nitrogen; NO_3_-N, nitrate-nitrogen; NH_4_-N, ammonium-nitrogen; MBN, microbial biomass nitrogen; TP, total phosphorus; AP, available phosphorus; TK, total potassium; AK, available potassium; TS, total sulfur; AS, available sulfur; AFe, available iron; TFe, total iron; CEC, cation exchange capacity. Download FIG S2, PDF file, 0.3 MB. Copyright © 2019 Jiao et al. 2019 Jiao et al. This content is distributed under the terms of the Creative Commons Attribution 4.0 International license.

**FIG 1:**
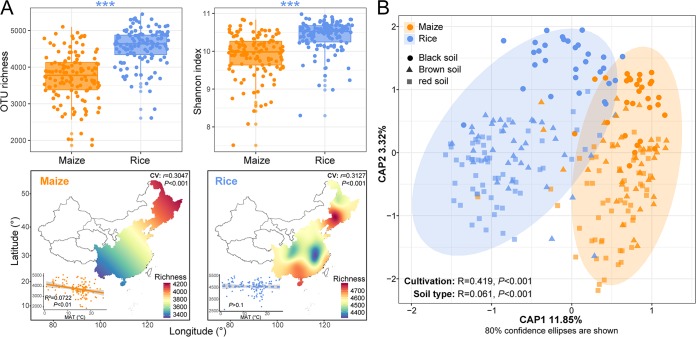
General patterns of bacterial *α*- and *β*-diversity in soil samples from maize and rice fields in eastern China. (A) Differences in *α*-diversity (operational taxonomic unit [OTU] richness and Shannon index) were estimated for maize and rice soils. Blue asterisks indicate that the *α*-diversity index was significantly higher in rice soils (***, *P* < 0.001; Wilcoxon rank sum test). Spatial distributions of OTU richness were mapped, and their associations with mean annual temperature (MAT) for each pairwise set of soil samples were estimated via linear least-squares regression analysis. Cross-validation (CV) of the maps was based on Pearson correlation between the predicted and observed values at each sampling site. (B) Constrained analysis of principal coordinates (CAP) showing the *β*-diversity patterns between maize and rice soils and among different soil types. Eighty percent confidence ellipses are shown around each group. Similarity values among the samples between maize and rice soils (cultivation) and among soil types (soil type) were examined via the ANOSIM test and are shown at the bottom of the plot. The constraints of the CAP model are cultivation and soil type. The CAP model and its first two axes and terms were significant (*P* < 0.01; by permutation tests).

We identified a total of 13,222,875 high-quality sequences, which were clustered into 32,107 operational taxonomic units (OTUs) based on 97% sequence similarity. First, we determined bacterial α-diversity, including OTU richness and Shannon index. The overall α-diversity indices were significantly higher in rice soils than maize soils (Fig. 1A). When we modeled the spatial distributions of OTU richness using a kriging interpolation method, maize soils showed a greater value at high latitudes than low latitudes; however, this trend was not observed in rice soils. In addition, significant and negative linear regressions were found between bacterial α-diversity and mean annual temperature (MAT) for maize soils but not rice soils. Second, we estimated bacterial *β*-diversity based on Bray-Curtis distance. Constrained analysis of principal coordinates (CAP; Fig. 1B) and nonmetric multidimensional scaling ordination (NMDS; see Fig. S1B in the supplemental material) showed that soil samples from maize and rice fields formed distinct clusters in the ordination space, with significant differences being found at taxonomic levels (analysis of similarities [ANOSIM]). In addition, we observed significant differences in bacterial community among different soil types (*P* < 0.001). The different distributions of bacterial taxa between maize and rice soils and among soil types are detailed in the supplemental material.

### Identifications and spatial atlases of core bacterial taxa.

**FIG 2:**
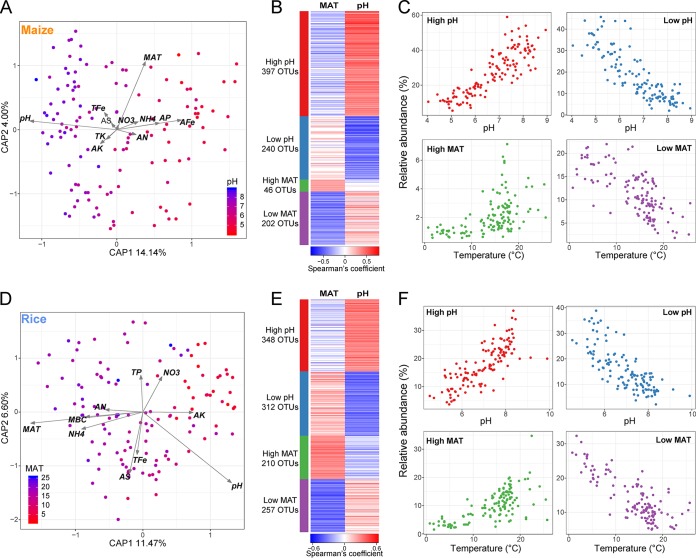
The main environmental drivers and ecological clusters of the core microbiota identified in soil bacterial communities from maize and rice fields. The main edaphic and climatic factors that influenced the bacterial community assemblage in maize (A) and rice (D) soils were identified by CAP analysis. Sample points are colored according to soil pH (maize panel) and mean annual temperature (MAT; rice panel). Color bars from red to blue represent values from small to large, respectively. Ecological clusters of the core bacterial taxa in maize (B) and rice (E) soils were explored by Spearman correlations between the relative abundance of each core bacterial taxon and the major environmental drivers (soil pH and MAT). Four ecological clusters (high pH, low pH, high MAT, and low MAT) were identified and are displayed as heat maps. Relationships between the relative abundance of the taxa assigned to each ecological cluster and their major environmental drivers in maize (C) and rice (F) fields are shown.

To identify the core microbiota, we selected the most abundant and ubiquitous OTUs in all soil samples. A total of 1,038 and 1,383 OTUs were denoted as the core bacterial taxa in maize and rice soils, respectively, accounting for 4.1% and 5.0% of all observed taxa. However, these taxa accounted for, on average, 67.5% and 68.5% of sequences across all samples from maize and rice fields, respectively (see Tables S1 and S2 in the supplemental material). These core taxa were mainly classified into *Proteobacteria*, *Acidobacteria*, *Actinobacteria*, and *Chloroflexi*. To limit colinearity effects between variables, we used variable clustering to assess the redundancy of environmental variables, and the variables with Spearman’s correlation coefficients of >0.6 were removed in the further analysis (see Fig. S3 in the supplemental material). To seek for the major environmental variables in shaping bacterial community composition, we performed a distance-based linear model and forward selection procedure. Soil pH and MAT were found to be the most important variables for bacterial community assemblage in maize and rice soils, respectively (Fig. 2A and D). Since each individual soil type occurs across a broad latitudinal range, we examined whether MAT is another important variable for bacterial *β*-diversity independent of soil type. The permutational MANOVA (ADONIS) analysis showed that MAT significantly affected the bacterial community in each soil type (see Table S3A in the supplemental material). Thus, we focused on these two variables when identifying the ecological preferences of the core bacterial taxa based on Spearman correlations. Since some taxa were simultaneously correlated with both pH and MAT, we selected the correlations with higher coefficients as the preferred ecological attributes for the particular taxa. In total, the core taxa were grouped into four ecological clusters sharing environmental preferences for (i) high pH, (ii) low pH, (iii) high MAT, and (iv) low MAT (Fig. 2; also see Tables S2 and S3 in the supplemental material). The strong relationships between environmental variables and relative abundance of the corresponding ecological clusters indicated reasonably well-defined ecological clusters (Fig. 2).

10.1128/mSystems.00313-18.4 FIG S3 Cluster analysis of the measured environmental variables in maize and rice fields. The analysis was performed and plotted using varclus in the Hmisc R package. Abbreviations of environmental variables are defined in the Fig. S2 legend. Download FIG S3, PDF file, 0.1 MB. Copyright © 2019 Jiao et al. 2019 Jiao et al. This content is distributed under the terms of the Creative Commons Attribution 4.0 International license.

10.1128/mSystems.00313-18.8 TABLE S1 List of core microbiota found in soil bacterial community of maize fields across eastern China. This list contains information on the relative abundance, ubiquity, and taxonomic identity of each taxon; the Spearman correlation coefficient (*P* value < 0.05) between the relative abundance of each core bacterial taxon and environmental predictors; and the ecological cluster to which it was assigned. Download Table S1, XLSX file, 0.1 MB. Copyright © 2019 Jiao et al. 2019 Jiao et al. This content is distributed under the terms of the Creative Commons Attribution 4.0 International license.

10.1128/mSystems.00313-18.9 TABLE S2 List of core microbiota found in soil bacterial community of rice fields across eastern China. This list contains information on the relative abundance, ubiquity, and taxonomic identity of each taxon; the Spearman correlation coefficient (*P* value < 0.05) between the relative abundance of each core bacterial taxon and environmental predictors; and the ecological cluster to which it was assigned. Download Table S2, XLSX file, 0.1 MB. Copyright © 2019 Jiao et al. 2019 Jiao et al. This content is distributed under the terms of the Creative Commons Attribution 4.0 International license.

10.1128/mSystems.00313-18.10 TABLE S3 (A) The permutational MANOVA (ADONIS) analysis showed that MAT significantly affected the bacterial community in each soil type. (B) Variation explained by bacterial *α*- and *β*-diversity indices in the regression models of soil multinutrient cycling index for maize and rice fields. Download Table S3, PDF file, 0.05 MB. Copyright © 2019 Jiao et al. 2019 Jiao et al. This content is distributed under the terms of the Creative Commons Attribution 4.0 International license.

**FIG 3:**
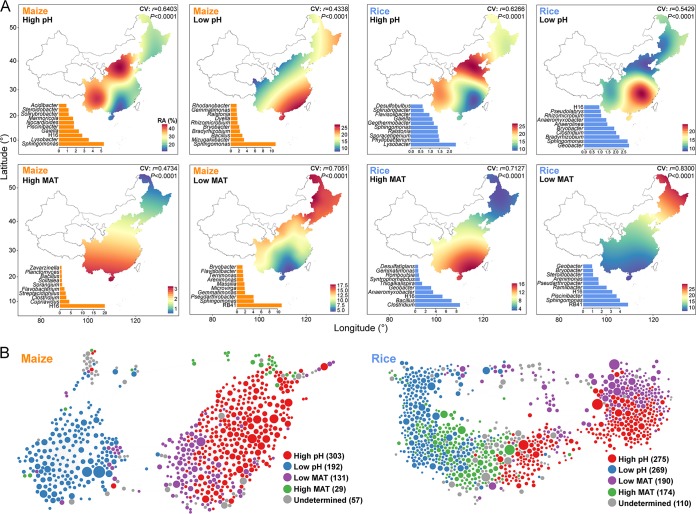
Habitat preferences of the core microbiota in soil bacterial communities in maize and rice fields. (A) A continental atlas of the core bacterial taxa in soil bacterial communities in maize and rice fields. The map shows the predicted spatial distributions of the relative abundance (RA%) of the four major ecological clusters of the core bacterial taxa sharing habitat preferences for high pH, low pH, high MAT, and low MAT. Cross-validation (CV) of the map was based on Pearson correlation between the predicted and observed values at each sampling site. (B) Network diagram with nodes (core bacterial taxa) colored according to each of the four major ecological clusters identified in maize and rice fields. The size of each node is proportional to the relative abundance, and the thickness of each connection between two nodes (edges) is proportional to the value of Spearman’s correlation coefficients.

To map the spatial distributions of the core taxa, we performed kriging interpolations on the relative abundance of each ecological cluster in maize and rice fields separately (Fig. 3A). Our maps provide estimates of the regions where we would expect the clusters of core bacterial taxa to be most abundant (Fig. 3A). The low- and high-MAT clusters were relatively abundant in low- and high-MAT regions, and the low- and high-pH clusters were particularly abundant in areas known for their low- or high-pH soils, respectively. Each of the ecological clusters identified included taxa belonging to multiple genera (Fig. 3A). We found that *Sphingomonas* was present in low-pH and high- and low-MAT clusters in both maize and rice fields. Meanwhile, *Lysobacter* and *Nocardioides* preferred high pH in maize fields. *Mizugakiibacter* and *Bradyrhizobium* were abundant in low-pH clusters in both maize and rice fields. In high-MAT regions, *Clostridium* and *Bacillus* were abundant in both maize and rice fields. RB41 preferred low-MAT environments in both maize and rice fields. In rice fields, *Geobacter* was consistent in low-pH as well as high- and low-MAT environments. Moreover, correlation network analyses were used to cross-validate whether bacterial taxa sharing similar habitats and environmental preferences tended to cooccur. All core taxa were included in the network analysis, while those taxa having no robust correlation relationships (Spearman’s correlation coefficients of >0.6 and false-discovery-rate-corrected *P* values of <0.01) with other taxa were lost during the generation of the networks. We found that nodes within the same ecological clusters (e.g., high pH, red nodes) were more connected (Fig. 3B).

### Ecological roles of core microbiota.

**FIG 4:**
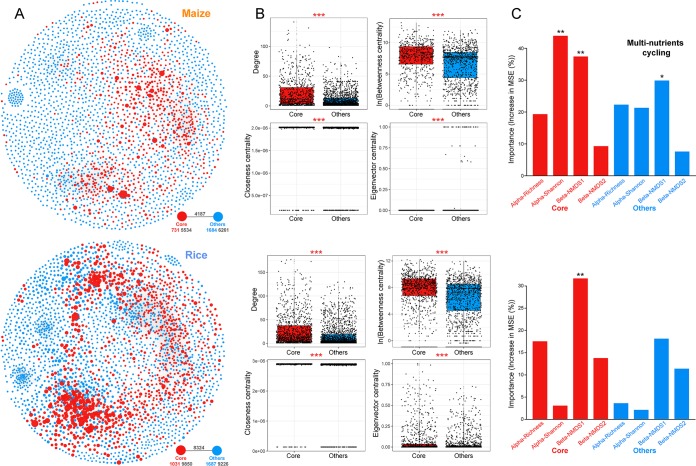
Ecological roles of the core microbiota in soil bacterial communities in maize and rice fields. (A) Metacommunity cooccurrence networks of core bacterial taxa in maize and rice soils based on Spearman’s correlation analysis. Cooccurrence networks are colored based on core and other taxa. A connection indicates a strong correlation (Spearman’s correlation coefficient of >0.6) and significance (false-discovery-rate-corrected *P* value of <0.01). The size of each node is proportional to the relative abundance of OTUs, and the thickness of a connection between two nodes (i.e., an edge) is proportional to the value of the Spearman correlation coefficient. The external associations (black numbers) among each subcommunity are displayed on the bottom right of each graph. Numbers in black below each node represent the inner associations of each subcommunity, and the number of nodes in each subcommunity is colored according to the categories. (B) Unique node-level topological features of core and other bacterial taxa, specifically the degree, betweenness, closeness, and eigenvector centrality. All features were significantly different between the two taxon groups based on Wilcoxon rank sum tests (***, *P* < 0.001). (C) Random forest mean predictor importance of bacterial *α*- and *β*-diversity indices for core and other subcommunities as variables for soil multinutrient cycling index. The accuracy importance measure was computed for each tree and averaged over the forest (5,000 trees). Significance levels are as follows: *, *P* < 0.05; **, *P* < 0.01. Alpha-Richness, OTU richness; Alpha-Shannon, Shannon index; Beta-NMDS1, the first axis of nonmetric multidimensional scaling analysis; Beta-NMDS2, the second axis of nonmetric multidimensional scaling analysis.

We then explored the ecological roles of the core microbiota in maintaining the connections between bacterial taxa. Metacommunity cooccurrence networks were established based on correlations for maize and rice soils, respectively. The network of maize soils consisted of 2,451 nodes (i.e., OTUs) and 15,982 edges; the rice soil network captured 2,718 nodes and 27,400 edges (Fig. 4A). The degree, betweenness, closeness, and eigenvector centrality of different subcommunities were significantly higher (*P* < 0.001; Wilcoxon rank sum tests) for the core taxa than for other taxa (Fig. 4B). To confirm these observations, we generated subnetworks for each subcommunity. The average degree, clustering coefficient, and graph density were all higher in the core subnetworks than in the other subnetworks. In contrast, the average path length and diameter were lower for the core subnetworks (Table 1).

**TABLE 1:** Topological features of different bacterial networks in the maize and rice soils

Category	Avg degree	Clustering coefficient	Avg path length	Diam	Graph density	Modularity
Maize						
Core	7.57	0.54	5.15	11.68	0.02	0.61
Others	3.72	0.44	10.30	17.05	0.01	0.81
Rice						
Core	9.55	0.54	4.51	7.83	0.02	0.60
Others	5.47	0.44	7.04	11.55	0.01	0.76
Whole	25.17	0.54	5.90	11.86	0.02	0.32
Maize	9.33	0.42	3.95	8.85	0.02	0.44
Rice	45.06	0.54	2.65	5.88	0.07	0.21

To disentangle the linkages between the core microbiota and soil multinutrient cycling in agricultural ecosystems, we applied a random forest (RF) analysis to identify the main microbial contributors to the soil multinutrient cycling index (see Fig. S4 in the supplemental material). We observed that the soil multinutrient cycling index for maize and rice soils was related to bacterial *α*-diversity and *β*-diversity, respectively. These observations were supported by multivariate regression analysis (see Table S3B in the supplemental material). Furthermore, we quantified the contributions of core and noncore subcommunities to the soil multinutrient cycling index (Fig. 4C). In both maize and rice soils, the bacterial diversity of the core microbiota, rather than other noncore taxa, made a major contribution to predicting the soil multinutrient cycling index. These results were supported by a multivariate regression analysis (Table 2), collectively pointing to strong associations between the core microbiota and belowground multinutrient cycling.

**TABLE 2:** Variation explained by bacterial *α*- and *β*-diversity indices of core and other bacterial subcommunities in the regression models of soil multinutrient cycling index for maize and rice fields

Diversity index	Soil multinutrient cycling index (%)	
	Maize	Rice
Core		
Alpha-Shannon	9.45	
Alpha-Richness	1.08	
Beta-NMDS1	4.69	9.72
Beta-NMDS2		2.16
Others		
Alpha-Shannon	3.04	
Alpha-Richness		
Beta-NMDS1		2.25
Beta-NMDS2		2.09
Total	20.16	16.21

10.1128/mSystems.00313-18.5 FIG S4 Disentangling bacterial predictors for soil multinutrient cycling index of maize and rice fields. RF mean predictor importance of bacterial *α*- and *β*-diversity indices as predictors for soil multinutrient cycling index. The accuracy importance measure was computed for each tree and averaged over the forest (5,000 trees). Significance levels are as follows: *, *P* < 0.05; **, *P* < 0.01. Alpha-Richness, OTU richness; Alpha-Shannon, Shannon index; Beta-NMDS1, the first axis of nonmetric multidimensional scaling analysis; Beta-NMDS2, the second axis of nonmetric multidimensional scaling analysis. Download FIG S4, PDF file, 0.1 MB. Copyright © 2019 Jiao et al. 2019 Jiao et al. This content is distributed under the terms of the Creative Commons Attribution 4.0 International license.

**FIG 5:**
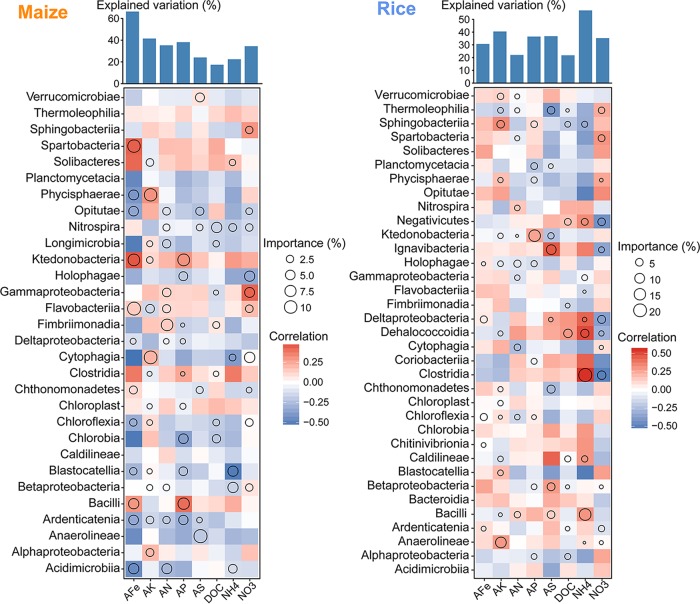
Potential biological contributions of the core microbiota at the class level to soil nutrient properties. Correlation and best multiple regression model for major taxonomic categories (class) of the core microbiota in maize and rice fields. Circle size represents the variable’s importance (i.e., proportion of explained variation calculated via multiple regression modeling and variance decomposition analysis). Colors represent Spearman correlations. AFe, available iron; AK, available potassium; AN, available nitrogen; AP, available phosphorus; AS, available sulfur; DOC, dissolved organic carbon; NH_4_, ammonium-nitrogen; NO_3_, nitrate-nitrogen.

Further, we used multiple regression modeling to evaluate the biological associations of the core microbiota at the class level with variations in soil available nutrient levels. We found that different bacterial classes contributed to the variations in nutrient levels in maize and rice soils (Fig. 5). For example, the *Fimbriimonadia* abundance was an important variable for predicting many nutrient levels in maize soils, including AN, AP, and DOC; however, this class only contributed to DOC level in rice soils. This result indicates the importance of *Fimbriimonadia* in soil nutrient cycling of maize fields, rather than rice fields. Other important variables for predicting soil nutrient properties in maize fields were the *Flavobacteriia* abundance for AFe, the *Cytophagia* and *Phycisphaerae* abundances for AK, and the *Bacilli* abundance for AFe and AP. In rice fields, the *Clostridia* and *Bacilli* abundances were the most important variables for predicting NH_4_, while the *Anaerolineae* and *Ktedonobacteria* abundances were predictors for AK and AP, respectively.

### Distinct distributions and cooccurrence patterns of maize- versus rice-enriched taxa.

**FIG 6:**
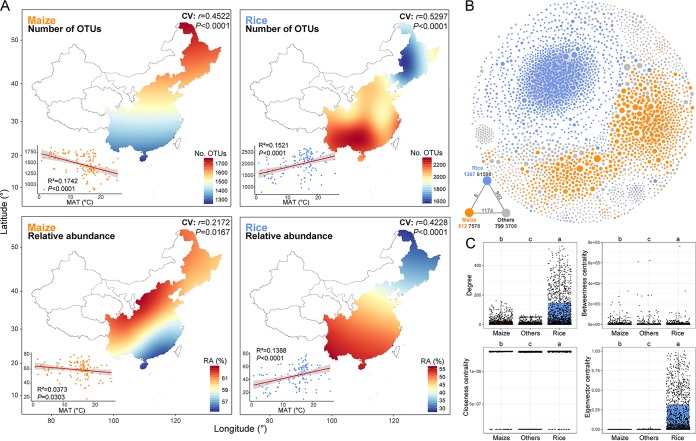
Distinct spatial distributions and cooccurrence patterns of maize- and rice-enriched bacterial taxa. (A) Relationships of the number of OTUs and the relative abundance of enriched taxa versus MAT were estimated for each pairwise set of soil samples via linear least-squares regression analysis. Cross-validation (CV) of the map was based on Pearson correlation between the predicted and observed values at each sampling site. (B) Metacommunity cooccurrence networks of bacterial taxa in maize and rice soils based on a Spearman correlation analysis. A colored cooccurrence network is shown for maize- or rice-enriched taxa. A connection indicates a strong correlation (Spearman’s correlation coefficient of >0.6) and significance (false-discovery-rate-corrected *P* value of <0.01). The size of each node is proportional to the relative abundance of the OTUs; the thickness of a connection between two nodes (i.e., an edge) is proportional to the value of Spearman’s correlation coefficient. The external associations (black numbers) among each subcommunity are displayed. The numbers in black below each node represent the inner associations of each subcommunity, and the numbers of nodes in each subcommunity are colored according to the categories. (C) Unique node-level topological features of different categories of taxa, specifically the degree, betweenness, closeness, and eigenvector centrality. Box plots that do not share a letter are significantly different (*P* < 0.05; multiple comparison by Kruskal-Wallis test). Maize, maize-enriched OTUs; Rice, rice-enriched OTUs; Others, nonsignificantly different OTUs.

To explore the distributions and cooccurrence patterns of bacterial communities, we identified significantly enriched OTUs for maize and rice soils, separately. In total, 3,484 and 6,170 OTUs were significantly higher in relative abundance in maize and rice soils, respectively. Across all sampling sites, the number and relative abundance of maize-enriched OTUs were higher in northern fields than southern counterparts, whereas the opposite trend was observed for rice-enriched OTUs. These results were verified by significant linear regression relationships, indicating distinct bacterial community assemblages between these two agricultural ecosystems (Fig. 6A). Next, we inferred a metacommunity cooccurrence network based on correlation relationships (Fig. 6B), capturing 74,966 associations among 2,978 bacterial OTUs. Rice- and maize-enriched OTUs clearly formed independent modules, and rice-enriched OTUs exhibited much closer interconnections.

To verify this observation, we examined the degree, betweenness, closeness, and eigenvector centrality of different groups of OTUs (Fig. 6C). The values of these topological features were significantly higher (*P* < 0.05) for rice-enriched OTUs than maize-enriched OTUs. Additionally, we generated subnetworks for rice- and maize-enriched communities and calculated a set of network-level topological features (Table 1). The average degree, clustering coefficient, and graph density were all higher in the rice subnetwork than the maize subnetwork, suggesting that rice-enriched OTUs were more connected. Additionally, the average path length and diameter were lower for the rice subnetwork, indicating closer relationships among rice-enriched communities.

## DISCUSSION

The soil microbial diversity promotes multifunctionality in natural terrestrial ecosystems (9, 10). Agricultural soils are typically Anthrosols influenced by human activity, and long-term agricultural land uses may alter soil microbial biogeographic distributions and microbially mediated nutrient cycling in agricultural ecosystems compared with natural ecosystems. Here, we revealed that the core microbiota play a major role in maintaining soil bacterial interactions while being strongly associated with belowground multinutrient cycling in agricultural fields across eastern China. We also built a continental atlas of the core bacterial taxa based on their habitat preferences, which shows the general spatial distributions of soil bacteria in agro-soils. The distinct biogeographic and ecological diversity patterns of bacterial communities determined the contributions of bacterial *α*-diversity and *β*-diversity to soil multinutrient cycling in maize and rice fields, respectively.

Long-term agricultural land uses can influence the soil environment, resulting in different microbial metabolic strategy and biogeography (11). Here, we observed an increasing trend in bacterial richness with increasing latitude in maize soils but not in rice soils. The latitudinal richness pattern we observed in maize fields is contrary to those most widely found in natural terrestrial ecosystems (17, 18). This discrepancy may be attributed to the differences between human-managed agricultural and natural terrestrial ecosystems. In addition, distinct irrigation management and agronomic practices could explain, at least in part, the different latitudinal richness patterns between maize and rice fields. Unlike dryland soils, waterlogged paddy soils provide a unique habitat as oxygen-limited conditions occur during constant flooding management (19). Due to dry-wet alternation, the bacterial communities in rice fields comprise both aerobic and anaerobic taxa; this explains the higher bacterial *α*-diversity we observed in rice fields than in maize fields. Among the environmental factors tested, soil pH had the biggest influence on the bacterial community structure in maize soils, while the bacterial assembly in rice soils was mainly correlated with MAT. These findings indicate the distinct diversity and assembly patterns of the soil bacterial community between maize and rice fields. Previous studies have demonstrated that soil microbial diversity promotes multifunctionality in terrestrial ecosystems, mainly natural dryland soils (9, 10). In the present work, we found that the soil multinutrient cycling index for maize and rice soils was related to bacterial *α*-diversity and *β*-diversity, respectively. In rice fields that are waterlogged during the growing season, metabolic cooperation via syntrophy between bacterial groups could play potential roles in the survival of the whole community under oxygen-limited conditions (20). This might explain the major contribution of bacterial *β*-diversity to soil nutrient cycling in rice fields. Together, our findings indicate the different performance of ecological diversity indices in the prediction of the soil multinutrient cycling index between maize and rice fields.

A better understanding of core (abundant and ubiquitous) soil bacteria is critical for us to manipulate soil bacterial communities for functional improvements. The global inventory of the dominant soil bacteria represents a small subset of phylotypes that account for almost half of the 16S rRNA sequences recovered from natural terrestrial ecosystems (14). In agricultural ecosystems, Walters et al. (21) performed a longitudinal study of maize fields, finding seven proteobacterial OTUs as the core microbiome in all samples (*n* > 4,800). Additionally, Edwards et al. (22) investigated the root-associated microbiome in rice fields across California’s Sacramento Valley, suggesting that the distribution of phyla in the root microbiome of rice may be generally applicable to terrestrial plants. In the present study, we conducted a larger latitudinal-scale survey of microbial communities and edaphic properties in soils sampled from adjacent pairs of maize and rice fields, which allowed for a direct comparison between maize- and rice-associated microbiomes. The core microbiota we identified in maize and rice soils accounted for almost 70% of all bacterial sequences, yet only ∼5% of total taxa. There were 814 core taxa present in both maize and rice fields, mainly belonging to *Sphingomonas*, *Bacillus*, *Lysobacter*, *Gaiella*, and *Ramlibacter*. The core taxa assigned to *Granulicella*, *Acidobacterium*, *Sphingobium*, and *Lechevalieria* were present only in maize fields, while the anaerobic genera *Anaerolinea*, *Sporacetigenium*, and *Syntrophorhabdus* were identified as the unique core taxa for rice soils. This result indicates the preferences of particular bacteria for a dry or waterlogged soil environment. Comparing our findings with the global inventory of dominant soil bacteria (14), we found that some of the core taxa in our agro-soils also occur in natural ecosystems, such as members of *Sphingomonas*, *Bacillus*, *Ramlibacter*, *Nocardioides*, *Flavisolibacter*, and *Bradyrhizobium.* Meanwhile, many core taxa identified in our agro-soils occur at low levels in natural terrestrial ecosystems, such as *Bryobacter*, *Lysobacter*, *Geobacter*, *Gaiella*, and *Thiobacillus*; these unique taxa may serve as indicator bacterial groups for agro-ecosystems.

In particular, we built a map for the spatial distributions of the core bacterial taxa at the continental scale. There were predictable environmental preferences for the core bacterial taxa in maize and rice soils, and their spatial distributions could be mapped by the continental atlas. Moreover, we revealed that the core bacterial taxa tended to cooccur with other taxa sharing a similar habitat preference. Habitat preferences of bacterial taxa are associated with their ecological attributes (23, 24). For example, we found the acid-tolerant *Bradyrhizobium* (25) preferred low-pH environments, whereas *Nocardioides*, generally isolated from alkaline environments (26), was abundant in the high-pH cluster. In addition, *Iamia*, *Lentzea*, and *Mesorhizobium* in the high-pH cluster and *Rhodoplanes* in the low-pH cluster were coincidentally observed in the previous work that defined the ecological clusters of high pH and low pH (14). These consistent lines of evidence strengthen the overall reliability of our predicted environmental preferences for the core taxa of soil bacteria in agro-soils. In particular, we defined high-MAT and low-MAT ecological clusters in agricultural fields for the first time. Interesting results were observed: for example, *Clostridium* and *Bacillus*, which can generate high-temperature-resistant endospores (27), were abundant in high-MAT regions; in contrast, RB41, *Sphingomonas*, and *Bryobacter* preferred low-MAT environments. Temperature has proven to be an important factor affecting soil microbial community structure and associated ecosystem functions (28–30). Our findings provide useful information for predicting the preferred environmental conditions (e.g., low or high pH and MAT) of specific bacterial taxa and enriching particular taxa *in vitro* and therefore increase the probability of their successful cultivation.

Given their ubiquity in a given environment, discovering the ecological roles of the core bacteria is critical to understanding the assembly and stability of bacterial communities (12). In the network analysis, topological features provide indicators for evaluating the roles of nodes (31, 32). High values of the topological features indicate the core and central position of a node in the network, whereas low values indicate a peripheral position (33, 34). In the present study, the higher values of topological features we observed for the core taxa indicate that members of the core microbiota were more often located in central ecological positions than other taxa. A more connected and complex subnetwork was therefore generated for the core microbiota. This may be related to the ubiquity of the core microbiota across different sites with greater environmental variation, which results in their occupation of a wider variety of ecological niches (33). Furthermore, we demonstrated the major contribution of the core microbiota, rather than other noncore taxa, to predicting soil multinutrient cycling. Thus, the core microbiota may play a vital ecological role in maintaining complex connections between bacterial taxa while being associated with belowground multinutrient cycling. In the present study, the potential involvement of the core taxa in various soil processes also implies their diverse functions in agro-ecosystems. For example, *Sphingomonas* and *Geobacter* have been reported to participate in herbicide metabolism (35) and the Fe biogeochemical cycle (36), respectively. *Clostridium* has been found to participate in C biogeochemical cycle processes such as organic matter decomposition (37). *Lysobacter*, which was found to prefer high-pH regions, displays powerful antibiosis effects (38). *Phyllobacterium* (an N_2_ fixer) and *Bacillus* (a P solubilizer) are known as plant growth-promoting bacteria (39, 40), while *Nocardioides* has been associated with *S*-triazine herbicide metabolism (41). The core microbiota are expected to be critical indicators of key soil processes worldwide, given the strong links between the distribution of bacterial phylotypes and their functional attributes across the globe (14).

Bacterial *β*-diversity exhibits strong habitat-specific patterns (42). Microbial ecological diversity patterns in rice and maize soils are likely not the same due to differences in oxygenation, irrigation management, and agronomic practices. In the current study, we observed distinct distributions of bacterial taxa that were significantly enriched in maize and rice fields; these enriched taxa showed negative and positive correlations with MAT, respectively, across the sampling sites. This suggests that soil bacteria in agricultural fields can adapt to and/or prefer different climatic conditions under various patterns of long-term crop cultivation or irrigation. Furthermore, cooccurrence network analysis revealed closer relationships among soil bacteria enriched in rice fields, which had a greater influence on other cooccurrences in the community, compared with maize-enriched species. We propose two possible reasons for our observation in rice fields: (i) the homogeneous conditions of waterlogged paddy soils increase niche overlap for more complex interactions between species (43, 44) and (ii) the greater diversity of aerobic and anaerobic taxa in rice fields is involved in various microbial processes, including nutrient cycling, resulting in dominant and broad ecological niches in the interaction network. These results provide a new perspective that distinct assembly patterns could drive bacterial cooccurrence patterns in maize and rice fields, resulting in different ecological positions in the network.

### Conclusions.

This study provides strong evidence to reveal the major ecological roles of the core microbiota in maintaining complex connections between bacterial taxa and their associations with belowground multinutrient cycling. The distinct biogeographic patterns of bacterial communities between maize and rice soils determine their different associations with soil multinutrient cycling in these two human-dominated agricultural ecosystems. Importantly, by identifying the habitat preferences of the core microbiota, we built a continental atlas for mapping the spatial distribution of soil bacteria, which helps predict the responses of agricultural ecosystems to anthropogenic disturbance. Our findings make it possible to predict how soil bacterial communities are likely to respond to current and future environmental changes and narrow down the research focus to a few hundred core taxa in the community, thus facilitating rapid and accurate forecasting of the ecological consequences of ongoing global environmental change.

## MATERIALS AND METHODS

### Sample collection.

The sampling regions of this study extend from 18.30°N to 48.35°N and from 87.61°E to 99.91°E (intervals of 18 to 3,689 km). We selected agricultural fields under long-term cultivation with maize and rice as representative dryland and wetland soils, respectively. To ensure appropriate spatial scale, sampling sites were selected from two adjacent maize and rice fields less than 5 km apart. In total, 133 locations were selected, comprising 118 paired sites, eight maize-only sites, and seven rice-only sites, giving 126 maize and 125 rice soil samples (see Fig. S1 in the supplemental material). During the planting season (July to September 2017), we sampled three plots (area = 100 m^2^) at each site and combined five soil cores per plot taken at a depth of 0 to 15 cm. Soil samples from the three plots at each site were mixed thoroughly to generate the final soil samples. All soil samples were delivered to the laboratory in sterile plastic bags on dry ice and sieved through a 2.0-mm mesh to remove plant debris and rocks. A portion of each soil sample was stored at 4°C for the analysis of edaphic factors (pH, CEC, OM, DOC, and T). AN, NO_3_-N, NH_4_-N, TP, AP, TK, AK, TS, AS, TFe, AFe, MBC, and MBN were measured using standard soil testing procedures (45). Aliquots of soil samples were stored at −20°C for subsequent DNA extraction. We obtained MAT and MAP values corresponding to the sampling site coordinates from the WorldClim database (www.worldclim.org). The soil types of samples were obtained from the China Soil Database (http://vdb3.soil.csdb.cn/), including black, brown, and red soil types from north to south (see Fig. S1 in the supplemental material).

### Illumina sequencing of 16S rRNA genes.

Total genomic DNA was extracted from soil samples using the MP FastDNA Spin kit for soil (MP Biomedicals, Solon, OH, USA) per the manufacturer’s instructions. Bacterial 16S rRNA gene V4-V5 hypervariable regions were PCR amplified using primers 515F (5′-GTG CCA GCM GCC GCG GTA A-3′) and 907R (5′-CCG TCA ATT CCT TTG AGT TT-3′) combined with adapter sequences and barcode sequences. Purified amplicons were sequenced by Novogene (Beijing, China) on a HiSeq2500 platform (Illumina Inc., San Diego, CA). Chimera detection and removal were accomplished using the Gold Chimera-Free reference database via the USEARCH option in the UCHIME algorithm. Sequences were split into groups according to taxonomy and assigned to OTUs at a 3% dissimilarity level using the UPARSE pipeline. OTUs with fewer than two sequences were removed, and representative sequence of each OTU was assigned to a taxonomic lineage by RDP classifier against the SILVA database.

### Quantifying the soil multinutrient cycling index.

Ecosystems perform multiple simultaneous functions and services (multifunctionality), rather than a single measurable process. Multiple-nutrient cycling is therefore the most important terrestrial ecosystem process for supporting human welfare (15). To quantify this vital provision, we selected eight measured available nutrient properties (DOC, AN, NO_3_, NH_4_, AP, AK, AS, and AFe) to construct a soil multinutrient cycling index analogous to the widely used multifunctionality index (9, 10, 46). These nutrient properties deliver some of the fundamental supporting and regulating ecosystem services (7, 9, 10), especially those essential for crop growth. For example, N and P are the nutrients that most frequently limit primary production in terrestrial ecosystems (47). NO_3_ is an important N source for both microorganisms and plants (47). AP is the main P source for plants and microorganisms, and it is linked to organic matter decomposition (47). K is the third essential macronutrient required by plants; it participates in a multitude of biological activities that maintain or improve crop growth, such as protein synthesis, enzyme activation, and photosynthesis (48). S and F are important mediators involved in biological electron transfer (49). To derive a quantitative soil multinutrient cycling index value for each site, we normalized (log-transformed as needed) and standardized each of the eight nutrient properties using the Z-score transformation. These standardized ecosystem functions were then averaged to obtain this index (10). We used this method to quantify soil multinutrient cycling because it is a straightforward and interpretable measure of a community’s ability to sustain multiple functions simultaneously (7, 9, 10).

### Statistical analyses.

To correct for sampling effort (number of analyzed sequences per sample), samples were rarefied at 27,812 sequences per sample for subsequent bacterial community analysis. Bacterial *α*-diversity (OTU richness and Shannon index) was calculated using QIIME (http://qiime.org/index.html). Bacterial *β*-diversity was estimated according to the Bray-Curtis distance between samples. An ANOSIM test was performed to determine significant differences in bacterial *β*-diversity between maize and rice fields and among soil types.

We respectively identified the core microbiota present in soil bacterial communities across maize and rice fields according to two criteria: (i) we selected highly abundant OTUs that were in the top 10% in terms of relative abundance across all samples and (ii) we retained ubiquitous OTUs that were present in more than 80% of all soil samples. These two criteria considered OTUs that were abundant and ubiquitous across different soil samples (14, 50). To limit colinearity effects between variables, variable clustering was used to assess the redundancy of environmental variables. The analysis was performed and plotted using the varclus procedure in the “Hmisc” R package. To test the significance and importance of the environmental variables for *β*-diversity, we used a distance-based linear model and forward selection procedure based on the Bray-Curtis distance matrix by estimating the proportion of variance explained (*R*^2^). These results were displayed by CAP analysis. To test the potential roles of soil pH and MAT in bacterial community assemblage, we clustered the core bacterial taxa into different ecological preferences based on these two environmental variables. We conducted Spearman correlations to identify groups of core taxa with shared habitat preferences. Since some taxa were simultaneously correlated with both pH and MAT, we selected the correlations with higher coefficients as the preferred ecological attributes for the particular taxa. We used a heat map to visualize our ecological clusters.

To build predictive maps of the spatial distributions of the core bacterial taxa, we used a kriging interpolation method to estimate the relative abundance of each ecological cluster in maize and rice fields, respectively. This analysis was performed using the “automap” package in R, which automates the interpolation process by automatically estimating a semivariogram and performing kriging. We cross-validated our maps using autoKrige.cv in the “automap” package. We extracted the predicted relative abundances of each cluster for the selected soil samples and correlated them with the observed values at the corresponding sites based on Pearson correlation analysis. The Pearson correlation coefficient and *P* value were shown in the map. We did not apply co-kriging interpolation that takes account of the influence of environmental variables, because (i) we wished only to display the general spatial distribution patterns of the microbial variables and (ii) high-resolution information on most of the soil variables was unavailable in agricultural fields at the continental scale (34).

The associations between microbial contributors and soil multinutrient cycling were evaluated by an RF analysis (10, 51). Bacterial *β*-diversity indices were quantified using the two axes of an NMDS analysis (52), including NMDS1 and NMDS2. In the RF models, bacterial *α*-diversity indices (OTU richness and Shannon index) and *β*-diversity indices (NMDS1 and NMDS2) served as predictors for the soil multinutrient cycling index. We then used a combined multiple regression model and variance decomposition analysis to validate the observations of RF analysis. In addition, bacterial diversity indices of the core and other subcommunities served as predictors for the soil multinutrient cycling index to estimate the contributions of the core microbiota to ecosystem functions. We also applied the multiple regression model with variance decomposition analysis to estimate the importance of the core microbiota at the class level for explaining each soil nutrient property. Class-level information was used in these analyses because information on microbial functional traits has become increasingly available at this taxonomic level and class-level taxa allowed us to infer general patterns in the role of microbial community composition in predicting soil nutrient properties (53).

To identify significantly enriched bacterial taxa in maize and rice soils, we applied a paired *t* test of the relative abundance of each OTU between different sample groups. OTUs of significantly higher abundance in maize samples were grouped as maize-enriched OTUs, whereas those with a significantly higher abundance in rice samples were categorized as rice-enriched OTUs, and those with no significant differences in relative abundance were categorized as “Others.” The differences obtained from paired *t* test results were verified to be significant by the Kruskal-Wallis test. Cooccurrence networks were constructed for bacterial communities among all soil samples. To reduce rare OTUs in the data set, we removed OTUs with a relative abundance of <0.01%. The Spearman correlation between each two OTUs was estimated. Robust correlations with Spearman correlation coefficients of >0.6 and false-discovery-rate-corrected *P* values of <0.01 were identified to construct networks in which each node represents one OTU and each edge represents a strong and significant correlation between two nodes. Six network-level topological features of the different networks were estimated for a set of metrics: average path length, average degree, graph density, clustering coefficient, diameter, and modularity. In addition, four node-level topological features were calculated for each node: degree, betweenness, closeness, and eigenvector centrality. Networks were visualized using the interactive Gephi platform.

### Data availability.

The soil bacterial data set is deposited in the Genome Sequence Archive (54) at the BIG Data Center (55), Beijing Institute of Genomics, Chinese Academy of Sciences, under the accession number PRJCA001121 and is publicly accessible at http://bigd.big.ac.cn/gsa.

10.1128/mSystems.00313-18.1 TEXT S1 Supporting information. Download Text S1, PDF file, 0.1 MB. Copyright © 2019 Jiao et al. 2019 Jiao et al. This content is distributed under the terms of the Creative Commons Attribution 4.0 International license.

10.1128/mSystems.00313-18.6 FIG S5 Variation in the relative abundance of dominant bacterial genera between maize and rice soils. All show significant group differences based on Wilcoxon rank sum test (*P* < 0.05). Download FIG S5, PDF file, 0.3 MB. Copyright © 2019 Jiao et al. 2019 Jiao et al. This content is distributed under the terms of the Creative Commons Attribution 4.0 International license.

10.1128/mSystems.00313-18.7 FIG S6 Canonical discriminant analysis comparing soil types against bacterial taxon loadings based on genera with relative abundance of >0.5% in maize and rice fields, respectively. Arrows represent the degree of correlation between each taxon and each treatment as a measure of the predictive discrimination of each treatment. Circles represent the canonical group means and 80% confidence interval for each class. Download FIG S6, PDF file, 0.2 MB. Copyright © 2019 Jiao et al. 2019 Jiao et al. This content is distributed under the terms of the Creative Commons Attribution 4.0 International license.
