# Supplementary figures and images for: Core Microbiota in Agricultural Soils and Their Potential Associations with Nutrient Cycling
Source: mSystems. 2019 Mar 26;4(2):e00313-18. doi: 10.1128/mSystems.00313-18 (PMC6435817; doi:10.1128/mSystems.00313-18)

**Figure S1**

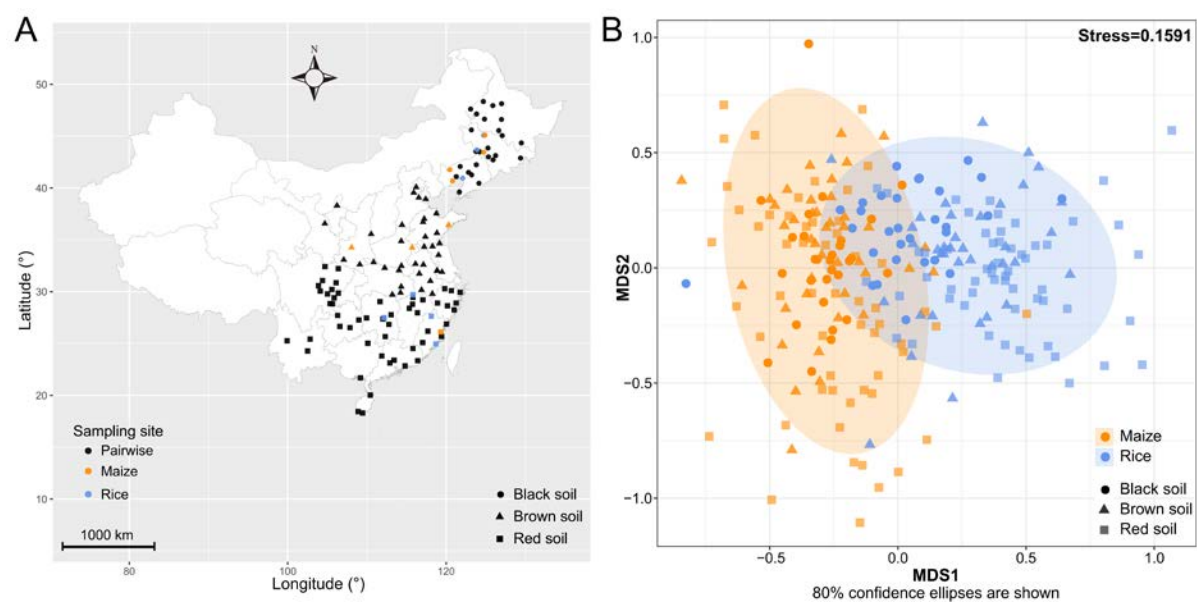

Supplement: FIG S1 [file mSystems.00313-18-sf001.pdf]

**Figure S2**

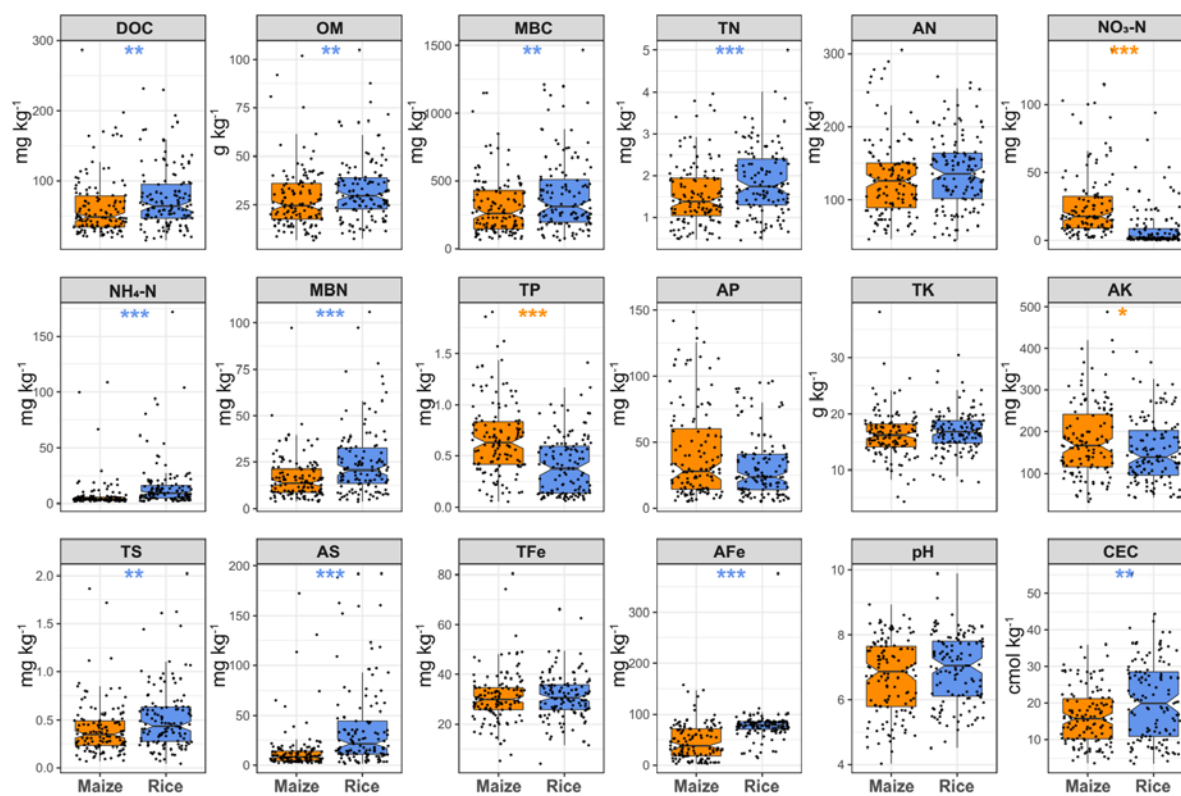

Supplement: FIG S2 [file mSystems.00313-18-sf002.pdf]

**Figure S3**

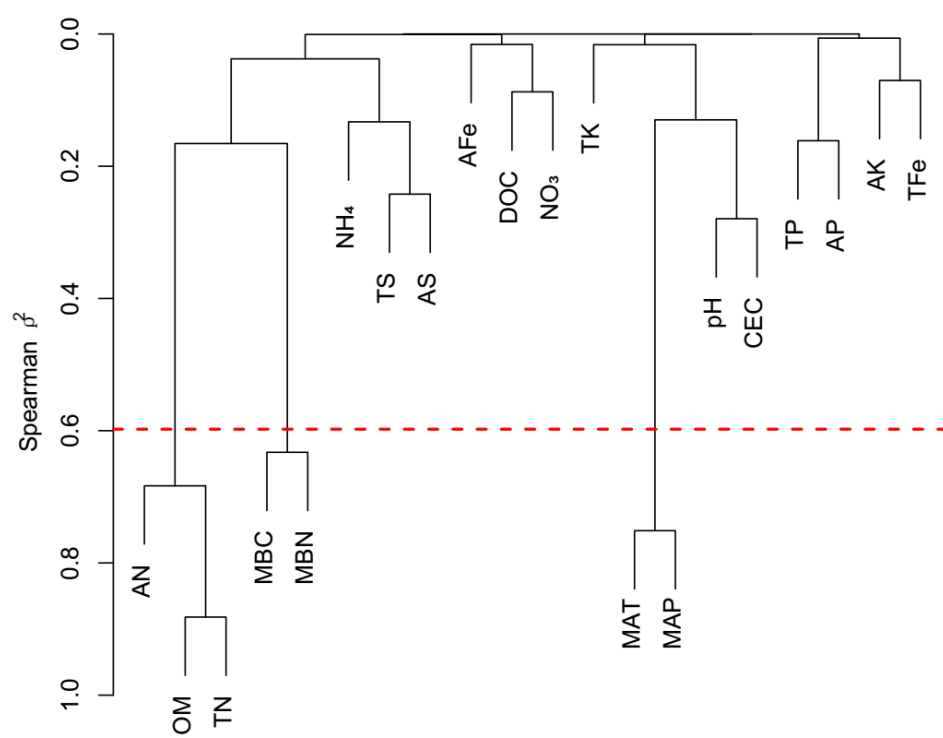

Supplement: FIG S3 [file mSystems.00313-18-sf003.pdf]

Figure S4

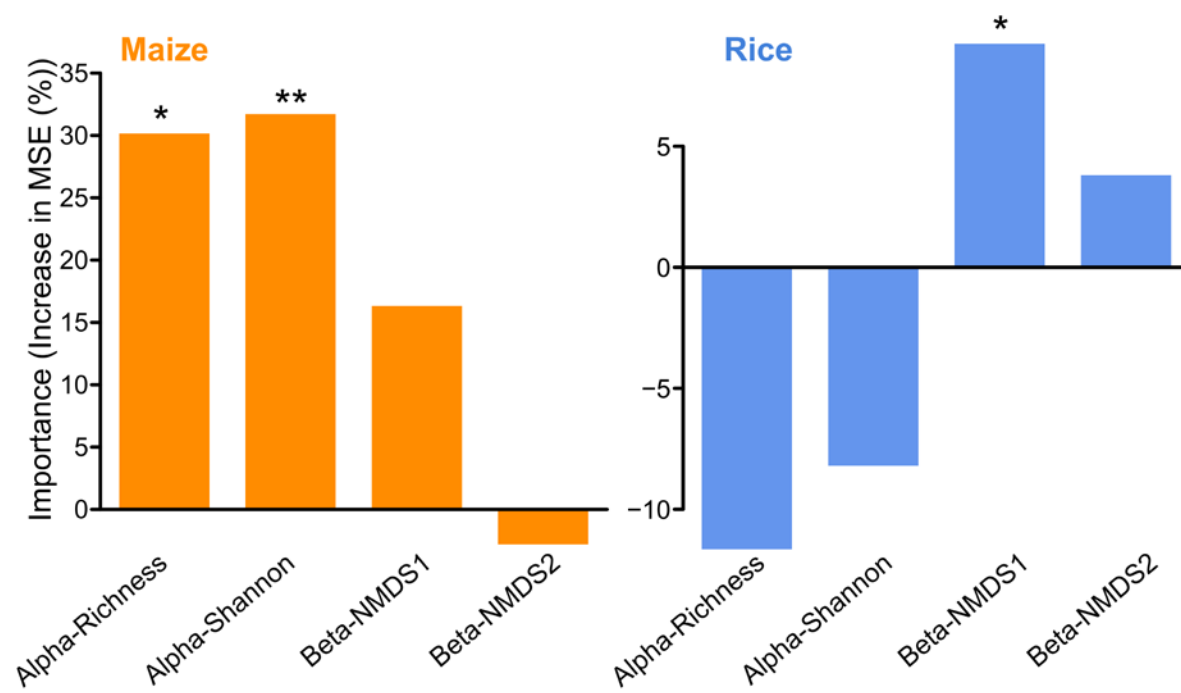

Supplement: FIG S4 [file mSystems.00313-18-sf004.pdf]

Figure S5

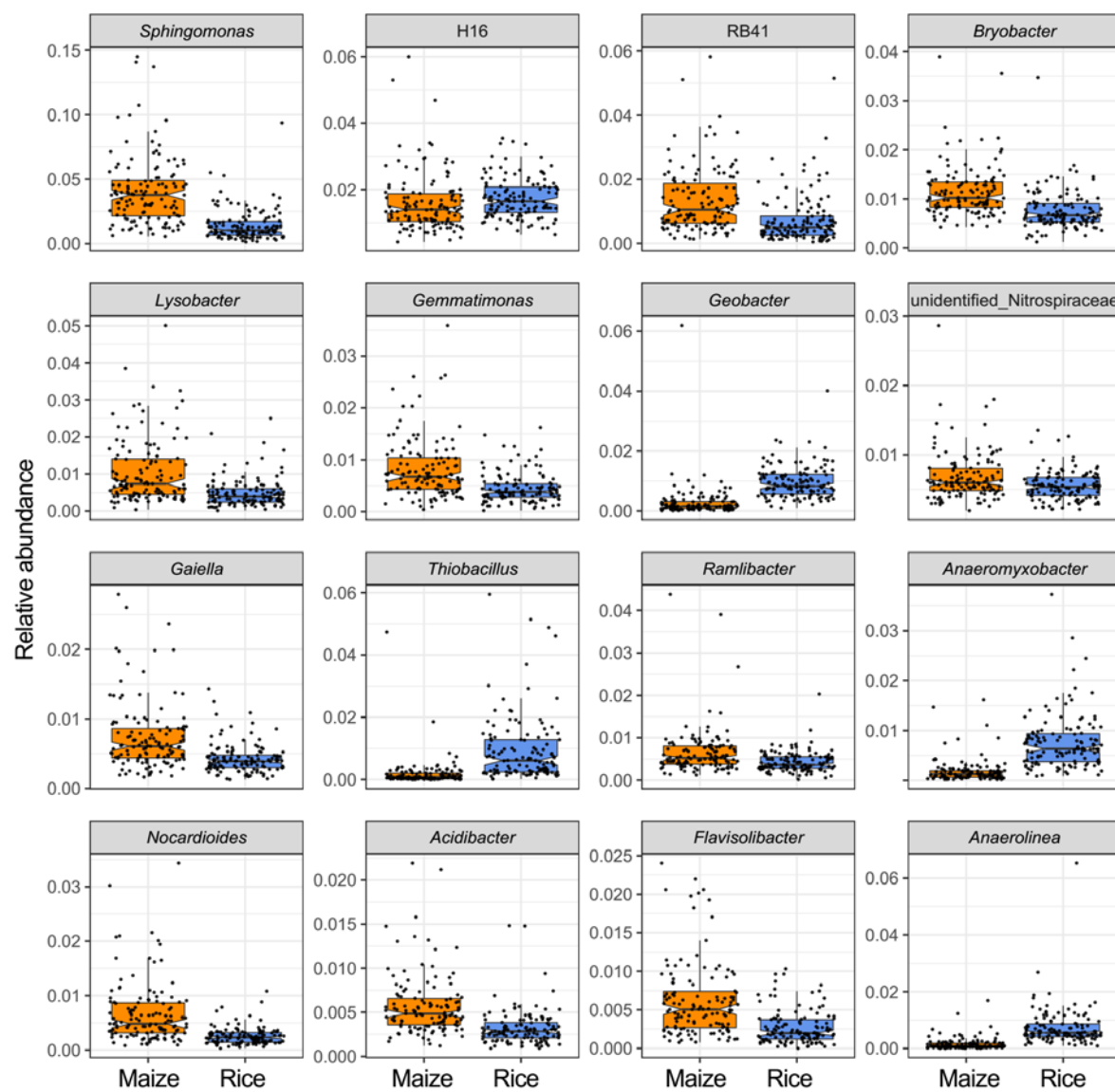

Supplement: FIG S5 [file mSystems.00313-18-sf005.pdf]

Figure S6

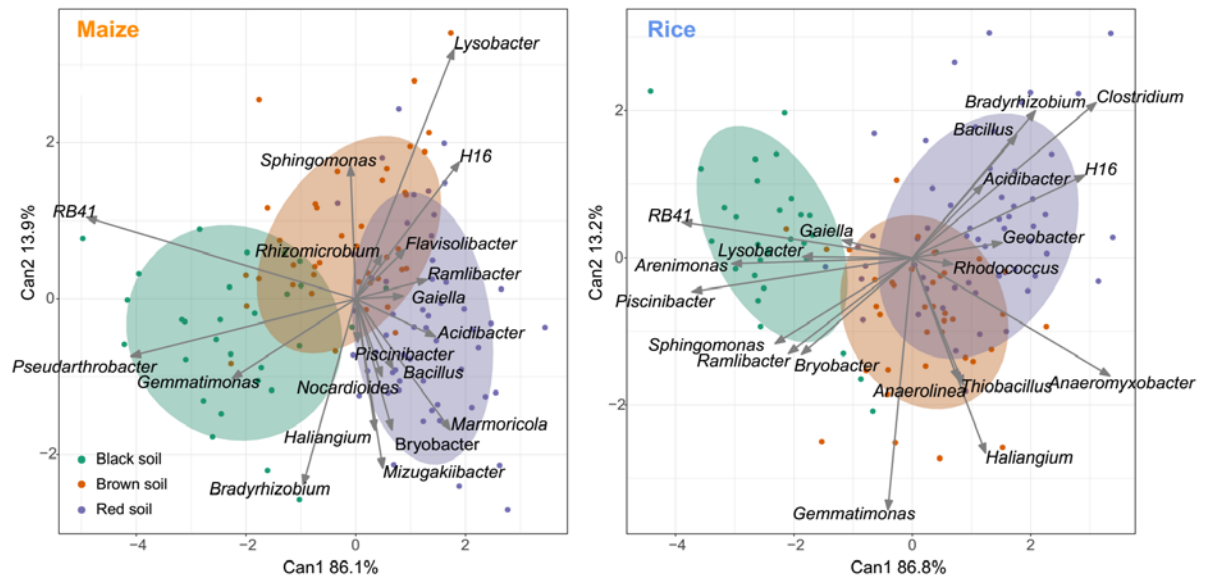

Supplement: FIG S6 [file mSystems.00313-18-sf006.pdf]
