# Supplementary material for: Core Microbiota in Agricultural Soils and Their Potential Associations with Nutrient Cycling
Source: mSystems. 2019 Mar 26;4(2):e00313-18. doi: 10.1128/mSystems.00313-18 (PMC6435817; doi:10.1128/mSystems.00313-18)
Supplement: TABLE S3 [file mSystems.00313-18-st003.pdf]

Table S3A The Permutational MANOVA (ADONIS) analysis showed that MAT significantly affected the bacterial community in each soil type.

Soil type	R^2	
	Maize	*Rice*
Black soils	0.0558*	0.0399*
Brown soils	0.0752***	0.0844***
Red soils	0.0578**	0.0441***

* *p* <0.05; ** *p* <0.01; *** *p* <0.001

Table S3B Variation explained by bacterial *α*- and *β*-diversity indices in the regression models of soil multinutrient cycling index for maize and rice fields.

Diversity index	Soil multi-nutrients cycling index	
	Maize	*Rice*
Alpha-Shannon	7.40%	
Alpha-Richness	9.37%	
Beta-NMDS1	4.93%	8.54%
Beta-NMDS2		1.78%
Total	21.70%	10.32%

NA, not statistically significant (*p* >0.05)
