# Supplementary material for: Core Microbiota in Agricultural Soils and Their Potential Associations with Nutrient Cycling
Source: mSystems. 2019 Mar 26;4(2):e00313-18. doi: 10.1128/mSystems.00313-18 (PMC6435817; doi:10.1128/mSystems.00313-18)
Supplement: TEXT S1 [file mSystems.00313-18-s0001.pdf]

Supporting information

Results and Discussion

The distributions of dominant bacterial genera (relative abundance >0.5%) were estimated in maize and rice soils (Supplementary **S5**). We found that paddy soils were mainly dominated by anaerobic such as *Geobacter*, *Anaeromyxobacter* and *Anaerolinea*, which are known to be involved in Fe, S, and C biogeochemical cycles (1). *Sphingomonas* and *Lysobacter* that dominate in maize fields are believed to be involved in antibiosis and herbicide metabolism (2, 3), and are beneficial to crop production and soil health. Paddy soils are submerged during the growing season, and oxygen is rapidly depleted at just a few millimeters beneath the water-soil interface (4), resulting in a specific environment that is favorable for anaerobic microorganisms. Indeed, remarkable differences in edaphic properties were observed, even though most soil samples were collected from pairs of adjacent maize and rice fields. Significantly higher of OM, DOC and TN, in rice fields indicated that long-term rice cultivation with dry-wet alternation could accumulate soil C and N. In addition, environmental factors involved in electron transfer, such as S and Fe, were significantly higher in anaerobic rice soils.

Given the significant impact of soil type on bacterial assembly, significant taxonomic differences among soil types were examined with a Canonical discriminant analysis based on genera with a relative abundance of >0.5% (Supplementary **Figure S6**). In maize fields, *Gemmatimonas*, and *Pseudarthrobacter* predominated in black soils; *Sphingomonas*, and *Lysobacter* were abundant in brown soils; while *Mizugakiibacter*, *Acidibacter*, and *Bacillus* were abundant in red soils. In rice fields, *Arenimonas*, *Piscinibacter*, and *Lysobacter* were

dominant in black soils; *Anaerolinea*, and *Haliangium* were abundant in brown soils; while Clostridium, *Bradyrhizobium*, and *Geobacter* were prominent in red soils. The spatial distributions of bacterial taxa are associated with their ecological characteristics, including physiological capabilities and habitat preferences (5, 6). Previous studies have demonstrated that soil type is an important determinant of microbial community assembly (7-9). In the present study, we also observed a strong impact of soil type on bacterial community assemblage in maize and rice soils, and most bacterial taxa displayed soil-type preferences. The specific bacterial taxa with soil type preferences were not the same in maize and rice fields, even though most soil samples were collected from two pairs of adjacent fields; this verified the distinct bacterial community assemblage between these two agricultural fields. The prominence of *Clostridium* (10), and *Acidibacter* (11) previously reported to be acid-tolerant, in red soils was attributable to low soil pH.

Reference:

1. **Cabezas A, Pommerenke B, Boon N, Friedrich MW.** 2015. *Geobacter*, *Anaeromyxobacter* and *Anaerolineae* populations are enriched on anodes of root exudate-driven microbial fuel cells in rice field soil. Environ Microbiol Rep **7:**489-497.
2. **Sorensen SR, Ronen Z, Aamand J.** 2001. Isolation from agricultural soil and characterization of a *Sphingomonas* sp. able to mineralize the phenylurea herbicide isoproturon. Appl Environ Microbiol **67:**5403-5409.
3. **Islam MT, Hashidoko Y, Deora A, Ito T, Tahara S.** 2005. Suppression of damping-off disease in host plants by the rhizoplane bacterium *Lysobacter* sp. strain SB-K88 is linked to plant colonization and antibiosis against soilborne peronosporomycetes. Appl Environ Microb **71:**3786-3796.
4. **Nicolaisen MH, Risgaard-Petersen N, Revsbech NP, Reichardt W, Ramsing NB.** 2004. Nitrification-denitrification dynamics and community structure of ammonia oxidizing bacteria in a high yield irrigated Philippine rice field. FEMS Microbiol Ecol **49:**359-369.
5. **Pointing SB, Chan YK, Lacap DC, Lau MCY, Jurgens JA, Farrell RL.** 2009. Highly specialized microbial diversity in hyper-arid polar desert. Proc Natl Acad Sci U S A **106:**19964-19969.
6. **Fierer N, Bradford MA, Jackson RB.** 2007. Toward an ecological classification of soil bacteria. Ecology **88:**1354-1364.

7. **Girvan MS, Bullimore J, Pretty JN, Osborn AM, Ball AS.** 2003. Soil type is the primary determinant of the composition of the total and active bacterial communities in arable soils. Appl Environ Microb **69:**1800-1809.

8. **Berg G, Smalla K.** 2009. Plant species and soil type cooperatively shape the structure and function of microbial communities in the rhizosphere. FEMS Microbiol Ecol **68:**1-13.

9. **Schreiter S, Ding GC, Heuer H, Neumann G, Sandmann M, Grosch R, Kropf S, Smalla K.** 2014. Effect of the soil type on the microbiome in the rhizosphere of field-grown lettuce. Frontiers in Microbiology **5**.

10. **Matthies C, Kuhner CH, Acker G, Drake HL.** 2001. *Clostridium uliginosum* sp nov., a novel acid-tolerant anaerobic bacterium with connecting filaments. Int J Syst Evol Micr **51:**1119-1125.

11. **Falagan C, Johnson DB.** 2014. Acidibacter ferrireducens gen. nov., sp. nov.: an acidophilic ferric iron-reducing gammaproteobacterium. Extremophiles **18:**1067-1073.
